# ZnO Hollow Quasi-Spheres Modified Screen-Printed Graphite Electrode for Determination of Carmoisine

**DOI:** 10.3390/mi14071433

**Published:** 2023-07-16

**Authors:** Sayed Zia Mohammadi, Somayeh Tajik, Farideh Mousazadeh, Elaheh Baghadam-Narouei, Fariba Garkani Nejad

**Affiliations:** 1 Department of Chemistry, Payame Noor University, Tehran P.O. Box 19395-3697, Iran; szmohammadi@pnu.ac.ir (S.Z.M.); elahehnarouei4@gmail.com (E.B.-N.); 2 Research Center of Tropical and Infectious Diseases, Kerman University of Medical Sciences, Kerman P.O. Box 76169-13555, Iran; f.garkani95@gmail.com

**Keywords:** ZnO hollow quasi-spheres, screen-printed graphite electrode, electrochemical sensor, carmoisine

## Abstract

Food colorants are important in food selection because they improve the gastronomic appeal of foods by improving their aesthetic appeal. However, after prolonged use, many colorants turn toxic and cause medical problems. A synthetic azo-class dye called carmoisine gives meals a red color. Therefore, the carmoisine determination in food samples is of great importance from the human health control. The current work was developed to synthesis ZnO hollow quasi-spheres (ZnO HQSs) to prepare a new electrochemical carmoisine sensor that is sensitive. Field emission-scanning electron microscopy (FE-SEM) and X-ray diffraction (XRD) have been used to analyze the properties of prepared ZnO HQSs. A screen-printed graphite electrode (SPGE) surface was modified with ZnO HQSs to prepare the ZnO HQSs-SPGE sensor. For carmoisine detection, the ZnO HQSs-SPGE demonstrated an appropriate response and notable electrocatalytic activities. The carmoisine electro-oxidation signal was significantly stronger on the ZnO HQSs-SPGE surface compared to the bare SPGE. Cyclic voltammetry (CV), linear sweep voltammetry (LSV), chronoamperometry (CHA), and differential pulse voltammetry (DPV) have been utilized to investigate the suggested protocol. The DPV results revealed an extensive linear association between variable carmoisine concentrations and peak current that ranged from 0.08 to 190.0 µM, with a limit of detection (LOD) as narrow as 0.02 µM. The ZnO HQSs-SPGE’s ability to detect carmoisine in real samples proved the sensor’s practical application.

## 1. Introduction

Food colorants are substances added to foods and drinks to enhance visual appeal. Therefore, the food processing industry uses a variety of edible colorants, including both natural and synthetic ones [1]. Food colorants are used to make food more visually appealing, and also to restore the original appearance of food whose color has been altered by processing, storage, packing, and distribution [2]. In the food industry, synthetic colorants, especially azo dyes, have been employed extensively to enhance food appearance and color due to their attractive color homogeneity, great water solubility, cheap production costs, low microbial contamination, and strong stability to light, oxygen, and pH [1]. Additionally, synthetic dyes are now widely applied in different industries, including those that deal with plastic, textiles, leather, detergents, and cosmetics. In addition, they are favored to enhance the organoleptic qualities of foodstuffs [3,4].

Griess originally discovered azo dyes, the most significant class of synthetic dyes, in 1865 [5,6]. Azo dyes have a strong water solubility due to their general chemical structure, which is made up of aromatic groups and an azo chromophore. Due to the presence of a double bond between two nitrogen atoms, this dye has an active functional group. Depending on how many azo functional groups they include, they can be mono-azo, di-azo, or poly-azo dyes [7]. The majority of these commercial dyes are mono-azo dyes [5]. Half of all dyestuffs that can be sold commercially contain azo dyes [5]. In other words, the most abundant family of synthetic dyes—azo dyes—are organic materials with the highest levels of production and consumption globally [8]. A lot of azo dyes are employed in the cosmetic, pharmaceutical, and leather sectors, particularly in the textile industry, because of their distinct physical and chemical qualities [5]. In addition, they are actively exploited as food colorings, medicine transporters, and have biological applications in addition to industrial uses [5]. The chromophore azo groups in azo dyes’ structures are transformed into extremely poisonous aromatic amines even though they have biological properties, such as being antibacterial and antiviral [5]. As a result, azo dyes and their metabolites are harmful to human health. In addition, excessive consumption of these substances is not recommended for both human health and food safety [3]. Some azo dyes are known to have negative effects, such as asthma and contact hypersensitivity. Also, azo dyes can cause food bigotry, hypersensitivity, and hyperactivity [3]. The European Union Regulation (EC) 1907/2006 forbids the use of some azo dyes due to the considerable risk they pose to the environment and human health [9]. Finally, it is crucial to use accurate and sensitive analytical techniques to control the determination of such significant substances. In common uses, such as food and beverages, carmoisine, a synthetic red dye with an azo bond in its molecular structure, provides a red to maroon shade [10].

Carmoisine was used as a coloring agent for jams and preserves for a long time, but most developed countries eventually outlawed it since it included the well-known carcinogen beta-naphthylamine [11]. In addition, reductive cleavage of azo groups produces an aromatic amine that is carcinogenic, like many azo dyes [12,13]. Many people also experience nettle rash, water retention, asthma, or drug intolerance as a result of the dye. In addition to increasing behavioral issues in children, such as hyperactivity, tantrums, and insomnia, large dosages of the dye can also cause coma, convulsions, somnolence, and even death [14]. Therefore, the amount of carmoisine in foods must be strictly managed, and it is crucial for both human health and the safety of food that carmoisine be detected quickly, sensitively, simply, and affordably.

A number of methodologies for quantifying synthetic food colors have been developed, including solid-phase extraction (SPE) [15], high-performance liquid chromatography (HPLC) [16], thin-layer chromatography (TLC) [17], spectrophotometry [18], capillary electrophoresis [19], solid-phase spectrophotometry [20], and fluorescence emission spectroscopy [21]. However, these methods have several drawbacks, including the need for specialized equipment, a laborious preparation/measurement procedure, as well as requiring a skilled operator for exact analysis [22,23]. Because electrochemical methods are capable of direct detection and do not require pretreatment procedures, they have recently received a lot of attention [24,25,26,27,28,29,30,31]. Furthermore, electrochemical methods are quick analytical methods that have the advantages of simplicity, great portability, and on-site detection ease [32,33,34,35,36,37]. 

Due to their reproducibility, reliability, affordability, and mass production, screen-printed electrodes (SPEs) became widely used in electrochemical sensing technology [38,39,40]. In electrochemical sensing technology, the selection of modified materials is crucial to achieve highly sensitive working electrodes for the detection of various chemicals [41,42,43,44,45]. Due to their new features, applications of nanoparticles have drawn a lot of attention recently and are now the subject of in-depth research in the disciplines of compound degradation and removal, catalysts, sensing, etc. [46,47,48,49,50,51,52,53,54,55]. Due to their enhanced electrochemical activities, surface modification employing nanomaterials has generated a great deal of interest to enhance the analytical performances of electrochemical sensors [56,57,58,59,60,61,62].

ZnO is a substance of substantial economic significance and is an essential semiconductor material. It has a high binding energy (60 meV), a bandgap of 3.37 eV, and is near in the ultraviolet spectral range [63]. Due to the numerous applications of nanoparticles based on their morphology, the shape and size control study of metal oxide nanoparticles (such as ZnO) has generated a great deal of scientific attention [63]. In recent years, ZnO hollow nanostructures are receiving a great deal of attention due to their unique electrical, optical, and surface properties, high surface-to-volume ratio, and lower density with good permeation [64,65,66,67].

The aim of this work was the synthesis of ZnO HQSs and the investigation of their application for modification of an SPGE as a sensing platform for the determination of carmoisine. The ZnO HQSs/SPGE sensor exhibited low LOD and high sensitivity for a wide linear range of carmoisine concentrations. In addition, good recovery values were obtained for the analysis of carmoisine in real samples.

## 2. Experimental

### 2.1. Chemicals and Solutions

All of the solutions utilized in the studies were prepared by utilizing ultra-pure water that had been purified by a Milli-Q^®^ system (Millipore, Burlington, MA, USA) with a resistivity of more than 18.2 MΩ cm. All of the chemicals have been purchased from Merck (Darmstadt, Germany) or Sigma-Aldrich (Steinheim, Germany). The phosphate buffer (0.1 M) was prepared by diluting the H_3_PO_4_ into ultra-pure water and adjusted to the desired pH with NaOH.

### 2.2. Equipments

A scanning electron microscope (MIRA3 SEM (Tescan, Brno, Czech Republic)) was utilized to analyze the surface morphology and structure of prepared ZnO HQSs. Cu Kα radiation (wavelength = 1.5406 Å) has been utilized for the XRD study with the Panalytical X’Pert Pro X-ray diffractometer (The Netherlands). Voltammetric measurements have been performed by using a potentiostat/galvanostat (PGSTAT (302N), Autolab, Eco-Chemie, Utrecht, The Netherlands). SPGEs (DropSens (DRP-110) Spain), consisting of a three-electrode system (WE = graphite working electrode, AE = graphite auxiliary electrode, and RE = silver pseudo-reference electrode, have been utilized. By utilizing a pH meter (model 827; Metrohm; Herisau; Switzerland), the pH values have been determined.

### 2.3. Synthesis of ZnO HQSs

With slight modifications, the ZnO HQSs were prepared in accordance with the previous work [68]. In the first step, 100 mL of polyethylene glycol (PEG) was used to dissolve 10 mmol (2.973 g) of Zn(NO_3_)_2_·6H_2_O under magnetic stirring (30 min) and ultrasonication (20 min) processes. After that, the mixed solution was added into a flask, and it was gradually heated to 160 °C. The process of refluxing was maintained at 160 °C with stirring for 6 h. The produced material was gathered by centrifugation, washed (several times) with ethanol and water, and then dried for 12 h at 60 °C. In the end, the white ZnO HQSs were prepared by calcining the dried material for four hours at 500 °C in a muffle furnace.

### 2.4. Preparation of ZnO HQSs/SPGE

For this purpose, 1 mg ZnO HQSs was dispersed (25 min) in 1 mL ultra-pure water. Following that, the working electrode was cast using 5 μL of ZnO HQSs suspension. To obtain the modified electrode, the solvent was evaporated. The ZnO HQSs/SPGE was prepared after the solvent was evaporated.

### 2.5. Preparation of Real Samples

To prepare the lemon juice sample, 10.0 mL of lemon juice was filtered using filter paper. Then, 2.0 mL of the filtered sample was diluted with 10.0 mL of phosphate buffer (0.1 M, pH = 0.7).

To prepare the powdered juice sample, 5 g of powdered juice was dissolved in 50 mL of deionized water (50 °C), and the solution was allowed to cool at room temperature. Then, the solution was diluted using 10 mL of phosphate buffer (0.1 M, pH = 0.7). In the next step, the diluted solution was filtered with a membrane filter (0.45 μm). 

## 3. Results and Discussions

### 3.1. Characterization of ZnO HQSs

**Figure 1:**
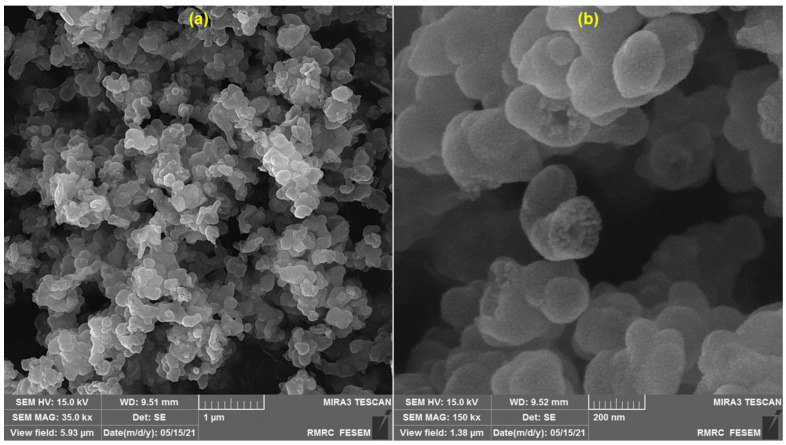
FE-SEM images of the as-prepared ZnO HQSs: a low-magnification FE-SEM image (1 µm) (**a**), and a high-magnification FE-SEM image (200 nm) (**b**).

FE-SEM was applied to study the structure and morphology of the as-prepared ZnO HQSs (Figure 1). In the low-magnification FE-SEM image in Figure 1a, the hollow structure cannot be detected, which shows that the ZnO quasi-sphere structures are prepared. The hollow structure of the ZnO HQSs, which have a mean diameter distribution of less than 200 nm, is clearly visible in the high-magnification FE-SEM image (Figure 1b).

**Figure 2:**
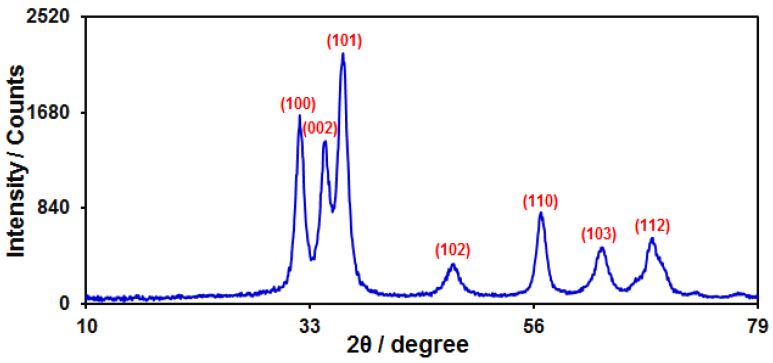
XRD pattern of ZnO HQSs.

The phase purity and crystallinity of ZnO HQSs were verified by XRD analysis. In Figure 2, the XRD pattern is displayed. The diffraction peaks located at 31.9°, 34.4°, 36.4°, 47.6°, 56.7°, 62.9°, and 68.1° correspond to diffraction from the (100); (002); (101); (102); (110); (103); and (112) planes of ZnO, respectively. All the peaks are indexed to the hexagonal ZnO with a wurtzite structure (JCPD No. 36-1451) [69,70].

### 3.2. Performance of the ZnO HQSs/SPGE Sensor for Carmoisine Determination

**Scheme 1:**
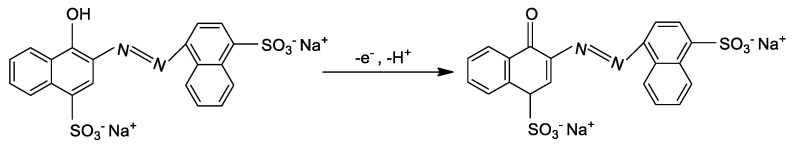
Schematic representation of carmoisine oxidation reaction on ZnO HQSs/SPGE sensor.

Investigating the electrocatalytic reaction of carmoisine at the ZnO HQSs/SPGE require adjusting the pH of the solution. As such, it also evaluated the effect of the aqueous solution’s pH value on the electrochemical activity of carmoisine. Due to this reason, the DPV was applied to examine the electrochemical reaction of carmoisine in 0.1 M phosphate buffer at various pH levels (3.0 < pH < 9.0) at the ZnO HQSs/SPGE surface. The results demonstrated that neutral conditions are preferable to acidic or alkaline conditions for the electrochemical oxidation of carmoisine. Thus, a pH of 7.0 (optimum pH) was considered for electrochemical investigations and measurements of carmoisine. The mechanism for oxidation of carmoisine on ZnO HQSs/SPGE is shown in Scheme 1.

**Figure 3:**
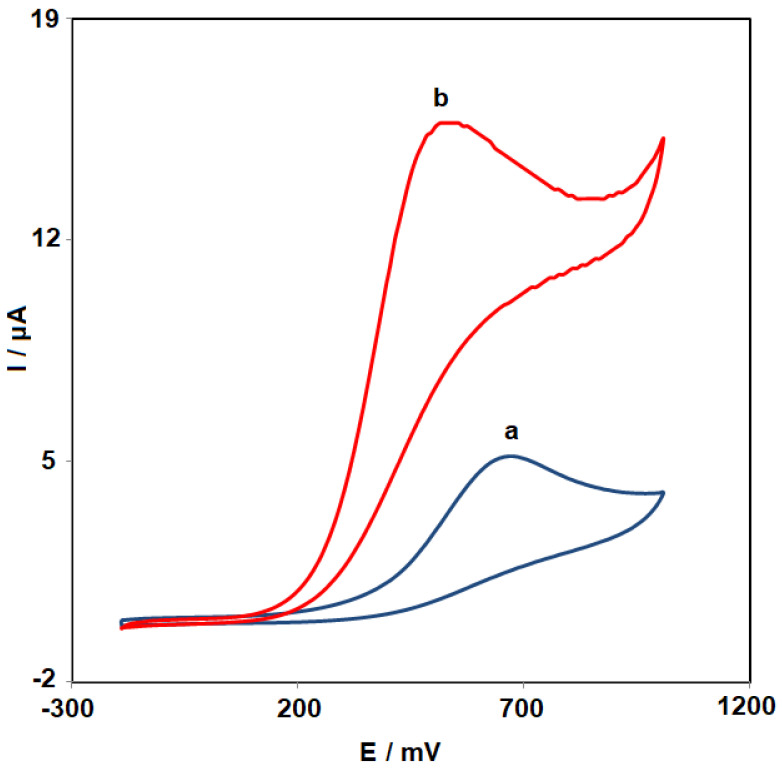
CVs of (a) unmodified SPGE and (b) ZnO HQSs/SPGE in the exposure to 100.0 µM carmoisine in 0.1 M phosphate buffer (pH = 7.0) at 50 mV s^-1^.

Figure 3 depicts the cyclic voltammograms of 100.0 µM carmoisine at unmodified SPGE (voltammogram a) and at ZnO HQSs/SPGE (voltammogram b). As can be seen, there was just one oxidation peak visible, indicating that the electrochemical reaction of carmoisine is an irreversible process. Also, the SPGE surface modification with ZnO HQSs had a significant effect on the Ep and Ip values. The Ep for carmoisine oxidation at the ZnO HQSs/SPGE was seen at 540 mV, which is roughly 130 mV more negative than that of unmodified SPGE, as can be seen. This shows that carmoisine oxidation happens more easily on the surface of the ZnO HQSs/SPGE. Additionally, a roughly threefold increase in Ip value at the surface of ZnO HQSs/SPGE is seen compared with the unmodified electrode. This result is attributed to the ZnO HQSs’ properties, such as their large surface areas, which boost electrocatalytic activity and raise Ip, which, in turn, increases the sensitivity of the sensor.

### 3.3. Effects of Scan Rate

**Figure 4:**
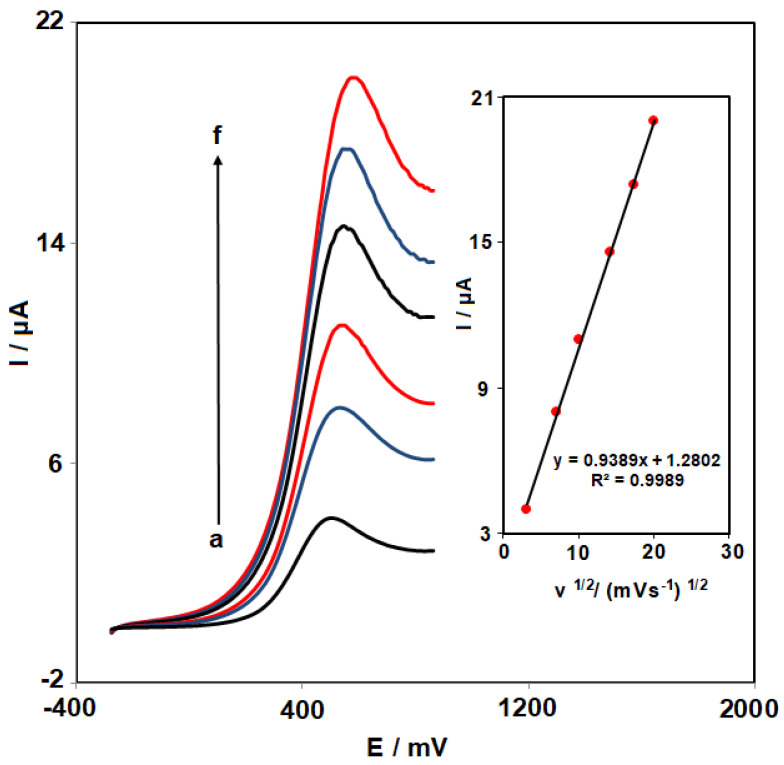
LSVs of ZnO HQSs/SPGE at various scan rates (a: 10; b: 50; c: 100; d: 200; e: 300; and f: 400 mV/s) in 0.1 M phosphate buffer (pH = 7.0) containing 50.0 µM carmoisine; Inset: changes in the Ip as a function of υ^1/2^.

The following stage involved determining how the scan rates affected the oxidation peak current of carmoisine (Figure 4). The recorded LSVs show that the oxidation peak current of carmoisine increased with the increase of scan rate. After recording the voltammograms at different scan rates, the plot of the current intensity (Ip) versus the square root of the scan rate (υ^1/2^) was drawn (Figure 4 (Inset)). According to the linearity of the resulting plot (regression equation: y = 0.9389x + 1.2802 with R^2^ = 0.9989), it was determined that the electrochemical reaction of carmoisine on the ZnO HQSs/SPGE is controlled through the diffusion phenomenon.

**Figure 5:**
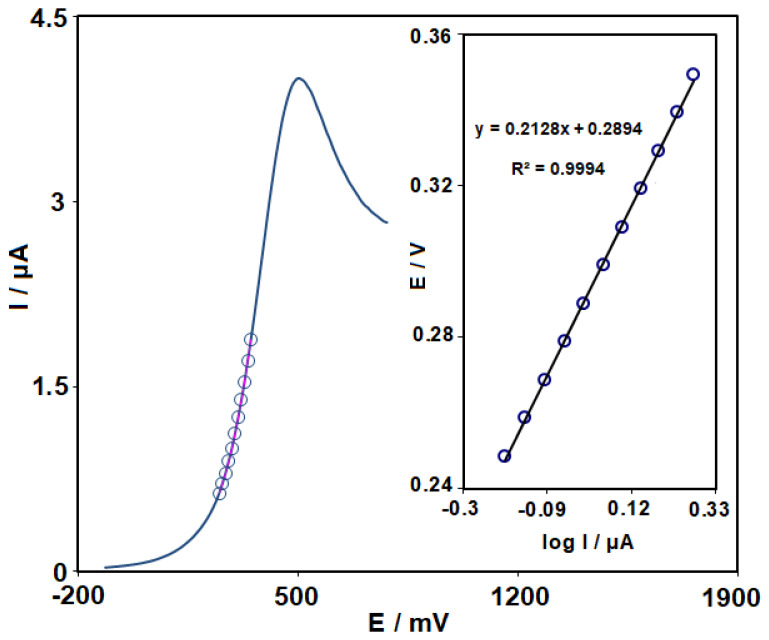
LSV captured for ZnO HQSs/SPGE in phosphate buffer (0.1 M; pH = 7.0) with carmoisine (50.0 µM) at the scan rate of 10 mV s^-1^; Inset: Tafel plot derived from LSV.

Then, a Tafel plot (Inset of Figure 5) has been drawn using data from the ascent of the I-E curve recorded at 10 mV s^−1^ (scan rate). The kinetic of electron transport between carmoisine and the ZnO HQSs/SPGE would have an impact on the Tafel area. Using Equation (1), the anodic transfer coefficient (α) can be obtained from the Tafel plot’s slope [71], as follows:

Ƞ = 2.3RT/(1 − α) nF log I + constant(1)

where: Ƞ (overpotential (V)), R (gas constant (J mol^−1^ K^−1^)), T (absolute temperature (K)), F (Faraday constant (C)), I (current (µA)), and n (number of electrons involved in the rate control step that is regarded as being 1). The value of α for carmoisine was determined as 0.72 using the Tafel slope (0.2128).

### 3.4. Chronoamperometric Measurements

**Figure 6:**
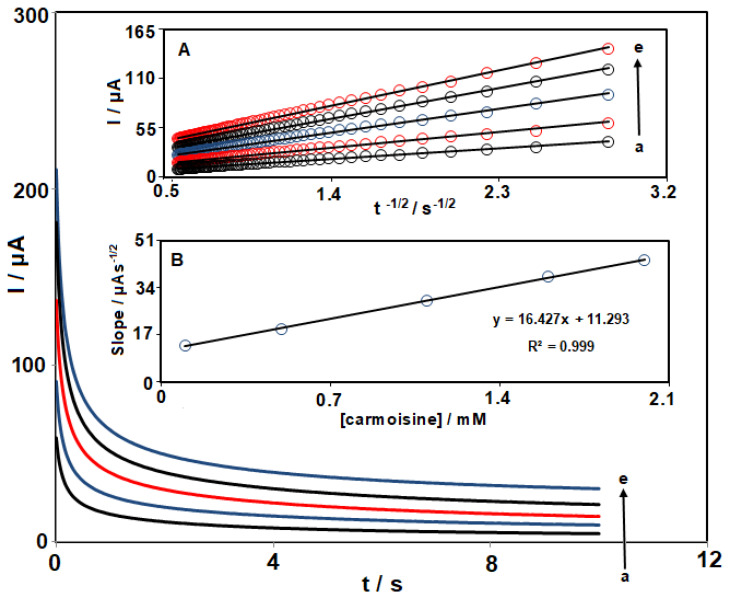
The chronoamperograms obtained for ZnO HQSs/SPGE in 0.1 M phosphate buffer (pH = 7.0; 0.1 M) containing various carmoisine concentrations (a: 0.1; b: 0.5; c: 1.1; d: 1.6; and e: 2.0 mM). I versus t^−1/2^ plots for recorded chronoamperograms (Inset (**A**)) and the slope of straight lines of graphs versus carmoisine concentrations (Inset (**B**)).

The potential of working electrode has been set at 590 mV in the following (Figure 6), to carry out the carmoisine chronoamperometric experiments on the ZnO HQSs/SPGE surface. The Cottrell equation (Equation (2)) [71] could be utilized to explain the current obtained by electrochemical reaction at the limited mass transport condition for an electroactive substance (carmoisine) that has a diffusion coefficient of D, as follows:

I = nFAD^1/2^ C_b_*π*^−1/2^t^−1/2^ (2)

Chronoamperometric measurements of carmoisine at various concentrations were carried out for this assessment. The plots of I against t^−1/2^ have been plotted (Figure 6A). The resulting straight lines’ slopes were then plotted against the carmoisine concentration (Figure 6B) in the following step. The Cottrell equation and final slope were used to determine the mean value of D for carmoisine, which has been determined to be 9.2 × 10^−5^ cm^2^ s^−1^.

### 3.5. Determination of Carmoisine by DPV

**Figure 7:**
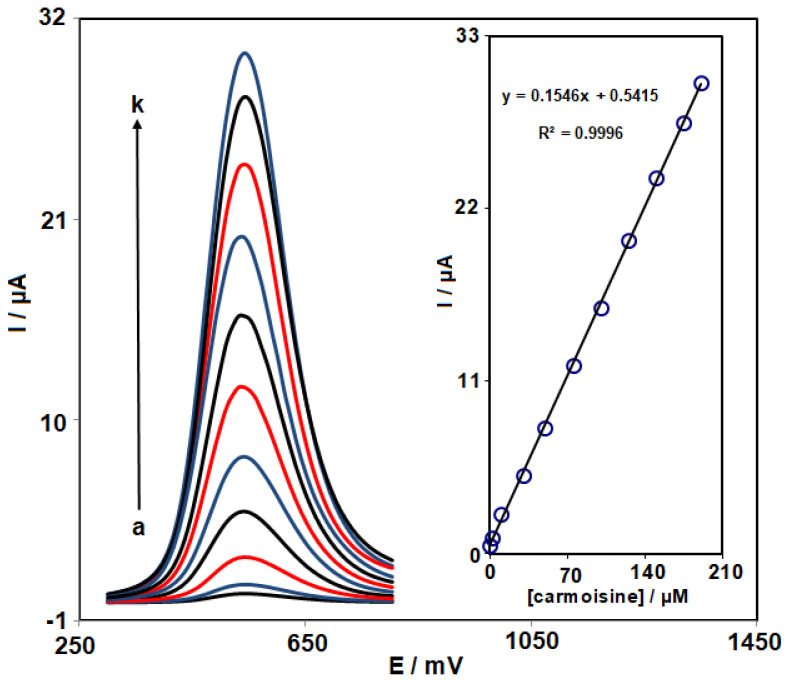
DPVs for ZnO HQSs/SPGE in 0.1 M phosphate buffer (pH = 7.0, 0.1 M) with various carmoisine concentrations (0.08, 2.0, 10.0, 30.0, 50.0, 75.0, 100.0, 125.0, 150.0, 175.0, and 190.0 μM); plot of peak current as a function of carmoisine concentration (Inset).

Given that DPV is more sensitive than other quantitative methodologies, this approach has been utilized to investigate the linear range and LOD. For DPV measurements, the following parameters have been employed: pulse amplitude (0.025 V), step potential (0.01 V), initial potential (300 mV), end potential (804 mV), and scan rate (50 mV s^−1^). For the purpose of this investigation, the DPVs of the ZnO HQSs were recorded for carmoisine with various concentrations. The data (Figure 7) show a linear relationship between the Ip and carmoisine concentration (in the range 0.08–190.0 µM) at the ZnO HQSs-SPGE surface. A detection limit of 0.02 µM for carmoisine has been subsequently determined. The obtained LOD value and linear range are compared with the reported data from different works (Table 1). As shown in Table 1, the performance of ZnO HQSs-SPGE is comparable to or better to that of the previously reported sensors. Moreover, the proposed sensor has some advantages including saving time, being cost effective, and the simplicity of electrode preparation, which proved that the proposed sensor has relatively good performance and can be used for the determination of carmoisine in food samples.

### 3.6. Repeatability, Reproducibility, and Stability

The repeatability, reproducibility, and stability of the ZnO HQSs/SPGE have been evaluated utilizing the DPV method. The reproducibility of ZnO HQSs/SPGE was remarkable, as shown by the 4.3% relative standard deviation (RSD) for the determination of 100.0 μM carmoisine on seven electrodes (ZnO HQSs/SPGE) constructed by using the same method. Seven repeated measurements of 100.0 µM carmoisine using the same electrode had an RSD of 3.5%, demonstrating the excellent repeatability of ZnO HQSs/SPGE. The modified electrode was kept for 24 days to investigate the stability of ZnO HQSs/SPGE. Findings show that the oxidation current of carmoisine reduced to 94.9% of its initial value after 24 days, confirming acceptable stability of electrode storage.

### 3.7. Interference Studies

The interference studies were carried out for a ZnO HQSs/SPGE sensor sensing 30.0 µM carmoisine in the presence of different species. The tolerance limit was considred as the concentration of foreign species that causes >±5% change in the peak current of target analyte. According to the findings, 50-fold of Na^+^, K^+^, Ca^2+^, Mg^2+^, NH_4_^+^, SO_4_^2−^, Cl^−^, and Br^−^, and 20-fold of glucose, alanine, tryptophan, and histidine did not show interference with the determination. 

### 3.8. Analysis of the Real Samples

By sensing carmoisine in powdered juice and lemon juice utilizing the standard addition method, the ZnO HQSs/SPGE’s applicability was evaluated. According to the data (Table 2), the recovery values were between 97.7% and 104.7%, and the RSD values (n = 5) were <3.5%, indicating that the developed ZnO HQSs/SPGE is reliable for the detection of carmoisine in real samples.

## 4. Conclusions

The present work was carried out to prepare a sensitive and reliable sensor (ZnO HQSs/SPGE) to detect trace amounts of carmoisine. The synthesized ZnO HQSs were characterized by XRD and FE-SEM techniques. The ZnO HQSs/SPGE was shown to have a distinctive electrochemical activity with a lower oxidation potential and higher oxidation current response to carmoisine than the unmodified electrode. The ZnO HQSs/SPGE sensor showed a linear relationship between the current and the concentration of carmoisine in a range between 0.08 and 190.0 µM and a low detection limit of 0.02 µM. Also, the ZnO HQSs/SPGE showed good repeatability, reproducibility, stability, and selectivity for the analysis of carmoisine. The effective capability of ZnO HQSs/SPGE was proven by detecting carmoisine in the real samples with satisfactory results.

## Figures and Tables

**Table 1:** Comparison of LOD and linear range for carmoisine with the previously reported works.

Electrochemical Sensor	Linear Range	LOD	Ref.
Silica/cetylpyridinium chloride (CPCl)/carbon paste electrode (CPE)	0.08 μM to 1.0 μM	0.01 μM	[4]
Cathodically pretreated boron-doped diamond electrode	0.0591 μM to 1.31 µM	0.007 µM	[10]
CdO/carbon nanotubes (CNTs)/ionic liquid (IL)/CPE	0.1 μM to 700.0 μM	40.0 nM	[72]
NiO-CNTs/ILCPE	70.0 μM to 650.0 μM	20 nM	[73]
Bismuth–chitosan/glassy carbon electrode (GCE)	1 µM to 41 µM	10 µM	[74]
Multi-walled carbon nanotubes (MWCNTs)/GCE	0.54 µM to 5.0 µM	0.11 µM	[75]
ZnO HQSs/SPGE	0.08 µM to 190.0 µM	0.02 µM	Present work

**Table 2:** Application of ZnO HQSs/SPGE for the carmoisine determination in the real specimens (n = 5).

Sample	Spiked Concentration (μM)	Found Concentration (μM)	Recovery (%)	RSD (%)
**Powdered juice**	0	3.4	-	3.2
	1.0	4.3	97.7	1.8
	2.0	5.5	101.8	2.3
	3.0	6.7	104.7	2.9
	4.0	8.3	98.8	2.1
**Lemon juice**	0	4.0	-	2.8
	1.0	5.1	102.0	3.5
	2.0	5.9	98.3	2.7
	3.0	7.3	104.3	2.0
	4.0	7.9	98.7	1.9
